# Association of difficulties in daily physical activities and handgrip strength with cancer diagnoses in 65,980 European older adults

**DOI:** 10.1007/s40520-023-02577-7

**Published:** 2023-10-27

**Authors:** Jonathan Martín-Cuesta, Joaquín Calatayud, José Casaña, Lee Smith, Shahina Pardhan, Guillermo Felipe López-Sánchez, Luis Suso-Martí, Ferran Cuenca-Martínez, Rubén López-Bueno

**Affiliations:** 1 Exercise Intervention for Health Research Group (EXINH-RG), Department of Physiotherapy, University of Valencia, Valencia, Spain; 2 National Research Centre for the Working Environment, Copenhagen, Denmark; 3 Centre for Health, Performance, and Wellbeing, Anglia Ruskin University, Cambridge, UK; 4 Vision and Eye Research Institute, School of Medicine, Faculty of Health, Education, Medicine and Social Care, Anglia Ruskin University, Cambridge, UK; 5 Physiotherapy Department, University of Valencia, Valencia, Spain; 6 Department of Physical Medicine and Nursing, University of Zaragoza, Zaragoza, Spain

**Keywords:** Cancer, Grip strength, Functional capacity, Limitations, Daily activities, Handheld dynamometer

## Abstract

**Background:**

People with cancer usually report physical deconditioning, which can limit daily activities.

**Aims:**

Our aim was to analyze associations between daily physical activities and handgrip strength with cancer diagnoses among European older adults.

**Methods:**

We used data from SHARE (a representative survey of individuals aged 50 years or older) wave 7, residing in 27 European countries and Israel. Participants self-reported difficulties in daily physical activities and cancer diagnoses, and handgrip strength was objectively assessed using a handheld dynamometer. Data were analyzed using binary logistic regression.

**Results:**

Overall, 65,980 participants (average age 67.6 years (SD = 9.4)) were analyzed. Having difficulties in any daily physical activity was significantly associated with higher odds of cancer diagnoses. Lower handgrip strength was significantly associated with cancer diagnoses among participants included in the first (adjusted odds ratio (AOR) = 1.27 [95%CI = 1.11–1.45]) and the second third (AOR = 1.15 [95%CI = 1.03–1.28]) when compared with participants from the last third in the final adjusted model.

**Discussion:**

Having difficulties in daily physical activities as well as lower levels of handgrip strength is positively associated with cancer diagnoses.

**Conclusion:**

Adults with difficulties lifting or carrying weights over 5 kilos or having difficulties in two or more activities showed critical associations with cancer diagnosis.

**Supplementary Information:**

The online version contains supplementary material available at 10.1007/s40520-023-02577-7.

## Introduction

Cancer is a leading cause of mortality globally [1]. In 2020, the International Agency for Research on Cancer estimated 19.3 million new cancer cases and 9.9 million cancer deaths occurred worldwide [2]. Furthermore, the number of cases and deaths is expected to increase rapidly over the next 2 decades as populations grow, age, and adopt lifestyle behaviors that increase cancer risk [2, 3].

The chance of developing cancer increases with increasing exposure to various non-modifiable (e.g., age or genetics) and modifiable risk factors (e.g. healthy lifestyle) [4]. According to the World Health Organization [5], 35% of deaths caused by cancer worldwide are due to potentially modifiable risk factors, for example, smoking, alcohol use, diet and physical inactivity. Among all these factors, physical inactivity (i.e. not meeting government physical activity guidelines for health) plays a fundamental role, since it is related to physical function and health [6, 7].

Due to advances in diagnosis, treatment and medical care, people with cancer are increasingly living longer [8]. Frequently, people with cancer report many symptoms, among them also exercise intolerance, and physical deconditioning, which can limit daily activities [9]. Being able to carry out normal daily activities is one of the determinants of the quality of life of cancer patients [10] and it has been suggested as a possible predictor of treatment outcome in cancer patients [11]. Thus, studies investigating the relationship between cancer diagnoses and daily activities are warranted. Cancer is associated with functional limitations, for example, a cross-sectional study [12] where physical function was objectively measured found an association between movement difficulty or complex activity limitations (for example, self-care limitations) with non-Hodgkin’s lymphoma and colorectal, ovarian and prostate cancer among US residents. Likewise, another cross-sectional study [13] among Canadian and Iranian patients reported that people who are diagnosed with and treated for cancer suffer great consequences in their daily life. However, to the best of our knowledge, studies investigating the association between different common daily physical activities and cancer diagnoses among the European population are scarce. This might be important since Europe has the highest incidence rates worldwide in several cancer types (e.g., colorectal, prostate, bladder or pancreas) [14]. Furthermore, most of the previous literature has a small sample with measures focused on general domains rather than specific daily activities (e.g., lifting or carrying weights, reporting difficulties pulling or pushing large objects, or handgrip strength), which could have greater impact in daily life and provide more specific information to implement focused preventive or rehabilitative strategies. Therefore, the aim of this cross-sectional study was to analyze the association between difficulties in daily physical activities and handgrip strength with cancer diagnoses among European older adults.

## Methods

### Study design and population

Data from Survey of Health, Ageing and Retirement in Europe (SHARE) wave 7, a representative survey from individuals aged 50 or over residing in 27 European countries and Israel, were retrieved [15]. Representativeness of SHARE waves is ensured using a multi-stage stratified sampling design, in which countries are divided into different strata based on geographical areas, and municipalities or zip codes within those strata are used as Primary Sampling Units (PSUs) with probability of being sampled proportional to their size [16]. The survey was carried out from February to November 2017 through computer-assisted personal interviews in the home of the respondents. Data from SHARE were collected using ex-ante harmonized interviews and provides calibrated weights to address the potential selection bias related to non-respondent errors. Moreover, new respondents are also added to compensate for such attrition. Respondents of both regular extended panels, and SHARELIFE survey along with a condensed regular panel were initially eligible for the study. Of the original sample, 10,612 participants with any missing values in the examined variables or not meeting the age criterium (i.e., ≤ 50 years) were excluded from the study (14%). The study followed the principles of the World Medical Declaration of Helsinki and was approved by the Ethics Committee of Research in Humans of the University of Valencia (register code 1,510,464).

### Previous or current cancer (exposure)

Previously experienced or current cancer was self-reported through the following question: “Has a doctor ever told you that you had/currently have any of the conditions on this card? With this we mean that a doctor has told you that you have this condition, and that you are either currently being treated for or bothered by this condition.”. The referred card included, among others, the following option: “Cancer or malignant tumors, including leukaemia or lymphoma, but excluding minor skin cancers”. Those participants selecting the aforementioned option were considered to have cancer diagnoses.

### Usual physical activities and handgrip strength (outcome)

Usual physical activities of the participants were assessed through the following question asked by the interviewers: “Please tell me whether you have any difficulty doing each of the everyday activities on this card. Exclude any difficulties that you expect to last less than 3 months”. The referred card included the following options: “Walking 100 m”, “Sitting for about 2 h”, “Getting up from a chair after sitting for long periods”, “Climbing several flights of stairs without resting”, “Climbing one flight of stairs without resting”, “Stooping, kneeling, or crouching”, “Reaching or extending your arms above shoulder level”, “Pulling or pushing large objects like a living room chair”, “Lifting or carrying weights over 10 pounds/5 kilos, like a heavy bag of groceries”, “Picking up a small coin from a table”, and “None of these”. An additional categorical variable considering those participants reporting difficulties with two or more of these physical activities was created. With regards to handgrip strength, that was measured twice with each hand using a handheld dynamometer (Smedley, S Dynamometer, TTM, Tokyo, 100 kg). Before the study period, interviewers participated in training sessions to learn a protocol for measuring handgrip strength. Participants were instructed to stand or sit with a 90° angle flexed elbow, neutral wrist position, and upper arm set in a vertical position against the trunk. Interviewers verbally encouraged participants with standardized instructions to perform the grip with maximum effort. The maximum value measured in one hand was considered as the maximum handgrip strength.

### Control variables

Based on prior research, self-reported sex, age, body mass index (BMI) derived from height and weight, and country of residence were considered as potential confounders in the main analyses [4, 17]. Additional analyses (Table S1) comprised a wider range set of confounders including multimorbidity, education, and other health-related variables such as physical inactivity, alcohol consumption, current smoking habit, and fruits and vegetables consumption with a substantial number of missing values. More details are provided in the Supplement.

### Statistical analyses

Complete-case statistical analyses were performed with Stata v16.1. Participants with cancer diagnoses anytime were compared in relation to physical movement difficulties, and handgrip strength using binary logistic regression. Using the confound user command option to identify the more accurate estimations for the model, this was finally adjusted for the following covariables: age, sex, body mass index, and country. To examine trends in the association between handgrip strength and cancer diagnoses, we categorized the variable using tertiles. Finally, to check the robustness of the associations between each of the exposition variables and the outcome, we also performed sensitivity analyses accounting for the complex survey-design, calibrated weights, and included other potential confounders such as comorbidity for an alternative complete-case model as well as education, physical inactivity, alcohol consumption, current smoking habit and fruits and vegetables consumption for an additional alternative model with imputed missing values (eTable1). Multiple imputation used a chained equation procedure comprising the covariates and the outcome variable and imputed five datasets. The level of statistical significance was set at *P* < 0.05.

## Results

A total of 65,980 participants (average age 67.6; SD 9.4; 55.6% women) years were included in the analyses. Of those, 4.8% declared having been diagnosed with cancer any time. Table 1 displays the basic characteristics of the study sample, including the percentage of participants reporting difficulties with physical activities, and categorized levels of handgrip strength. The most prevalent physical activity difficulties were stooping, kneeling, or crouching (30.6%), and climbing several flights without resting (28.5%).

**Table 1:** Study sample characteristics (*N* = 65,980)

Characteristic	Category	n (%)	Mean (SD)
Age			67.6 (9.4)
Sex	Men	29,264 (44.4)	
	Women	36,716 (55.6)	
Body mass index			27.2 (4.6)
Country	Austria	2713 (4.1)	
	Germany	3404 (5.2)	
	Sweden	2916 (4.4)	
	Spain	3667 (5.5)	
	Italy	3692 (5.6)	
	France	2962 (4.5)	
	Denmark	2989 (4.5)	
	Greece	2600 (3.9)	
	Switzerland	2214 (3.4)	
	Israel	1480 (2.2)	
	Belgium	4336 (6.6)	
	Czech Republic	3581 (5.4)	
	Poland	4189 (6.4)	
	Luxembourg	1003 (1.5)	
	Hungary	1334 (2.0)	
	Portugal	968 (1.5)	
	Slovenia	3138 (4.8)	
	Estonia	4099 (6.2)	
	Croatia	2133 (3.2)	
	Lithuania	1860 (2.8)	
	Bulgaria	1731 (2.6)	
	Cyprus	968 (1.5)	
	Finland	1888 (2.9)	
	Latvia	1503 (2.3)	
	Malta	1049 (1.6)	
	Romania	1890 (2.9)	
	Slovakia	1673 (2.5)	
Difficulties walking 100 m	No	59,392 (90.0)	
	Yes	6588 (10.0)	
Difficulties Sitting 2 h	No	59,884 (90.8)	
	Yes	6096 (9.2)	
Difficulties getting up from a chair	No	54,813 (83.1)	
	Yes	11,167 (16.9)	
Difficulties climbing one flight of stairs	No	57,985 (87.9)	
	Yes	7995 (12.1)	
Difficulties climbing several flights of stairs	No	47,164 (71.5)	
	Yes	18,816 (28.5)	
Difficulties stooping, kneeling, or crouching	No	45,810 (69.4)	
	Yes	20,170 (30.6)	
Difficulties reaching or extending arms above shoulder	No	60,818 (92.2)	
	Yes	5162 (7.8)	
Difficulties pulling or pushing large objects	No	56,029 (84.9)	
	Yes	9951 (15.1)	
Difficulties lifting or carrying weights over 5 kilos	No	52,677 (79.8)	
	Yes	13,303 (20.2)	
Difficulties picking up a small coin from a table	No	63,818 (96.7)	
	Yes	2161 (3.3)	
Several physical activity difficulties (2 or more)	No	43,080 (65.3)	
	Yes	22,900 (34.7)	
Handgrip (kg)			32.8 (11.6)
	Third 1	22,066 (33.4)	
	Third 2	23,063 (35.0)	
	Third 3	20,851 (31.6)	
Cancer diagnoses	No	62,839 (95.2)	
	Yes	3141 (4.8)	

Table 2 shows adjusted models for the association between difficulties with different daily physical activities and handgrip strength with cancer diagnoses. Reporting difficulties in each of the examined physical activities is significantly associated with higher odds of cancer diagnoses in both Model 1 and Model 2. Moreover, lower handgrip strength showed significant higher odds for cancer diagnoses among participants included in the first (AOR = 1.27 [95%CI = 1.11–1.45]) and the second third (AOR = 1.15 [95%CI = 1.03–1.28]) when compared with participants from the last third in the fully adjusted model (Model 2). Participants with difficulties lifting or carrying weights over 5 kg had the highest significant odds for cancer diagnoses in Model 2 (AOR = 2.04 [95%CI = 1.88–2.21]). Reporting difficulties in two or more physical activities ranked second for odds of cancer diagnoses in Model 2 (AOR = 1.95 [95%CI = 1.81–2.11]). Sensitivity analyses confirmed the robustness of the examined associations for difficulties reaching or extending arms above shoulder, climbing several flights without resting, difficulties pulling or pushing large objects, difficulties lifting or carrying weights over 5 kilos, and difficulties in two or more physical activities.

**Table 2:** Associations between difficulties in different daily physical activities, handgrip strength, and cancer diagnoses (*N* = 65,980)

		Model 1		Model 2	
		OR	95%CI	OR	95%CI
Difficulties walking 100 m	No	ref	Ref	ref	ref
	Yes	1.61	1.45–1.78	1.61	1.46–1.79
Difficulties sitting two hours	No	ref	Ref	ref	ref
	Yes	1.66	1.49–1.84	1.65	1.49–1.84
Difficulties getting up from a chair	No	ref	ref	ref	ref
	Yes	1.58	1.45–1.72	1.58	1.45–1.73
Difficulties climbing one flight of stairs	No	ref	ref	ref	ref
	Yes	1.51	1.37–1.66	1.51	1.37–1.67
Difficulties climbing several flights of stairs	No	ref	ref	ref	ref
	Yes	1.71	1.58–1.84	1.73	1.60–1.87
Difficulties stooping, kneeling, or crouching	No	ref	ref	ref	ref
	Yes	1.50	1.38–1.61	1.50	1.39–1.62
Difficulties reaching or extending arms above shoulder	No	ref	ref	ref	ref
	Yes	1.79	1.61–1.99	1.79	1.60–1.99
Difficulties pulling or pushing large objects	No	ref	ref	ref	ref
	Yes	1.89	1.73–2.06	1.91	1.75–2.09
Difficulties lifting or carrying weights over 5 kilos	No	ref	ref	ref	ref
	Yes	2.02	1.87–2.19	2.04	1.88–2.21
Difficulties picking up a small coin from a table	No	ref	ref	ref	ref
	Yes	1.48	1.26–1.74	1.47	1.25–1.73
Several movement difficulties (2 or more)	No	ref	ref	ref	ref
	Yes	1.91	1.77–2.07	1.95	1.81–2.11
Handgrip (kg) tertiles					
	Third 3	ref	ref	ref	ref
	Third 2	1.14	1.03–1.27	1.15	1.03–1.28
	Third 1	1.26	1.10–1.43	1.27	1.11–1.45

Model 1. Adjusted for age and sex Model 2. Adjusted for age, sex, body mass index, and country

*OR* Odds Ratio; *CI* Confidence Interval

## Discussion

The main findings of the study revealed that cancer diagnosis was positively associated with reporting difficulties in one or more relevant daily physical activities and negatively associated with handgrip strength. This association remained significant even when adjusting for relevant confounders. These results suggest that being diagnosed with cancer has a major impact on one's day-to-day life with difficulties in daily physical activities.

In a cross-sectional survey data analyzing a sample of 259,392 subjects [12], reporting at least 1 preexisting movement difficulty or complex activity limitations (including motor, sensory, emotional, or cognitive difficulties, and self-care, social, or work limitations) was associated with colorectal cancer, non-Hodgkin’s lymphoma, ovarian cancer, and prostate cancer. They also found that persons with preexisting disability generally had their cancers diagnosed at later ages than those without disability [12]. In another study with a sample of 66,641 women [18], authors found an association between movement difficulty or complex activity limitations and breast or cervical cancer. However, when other variables such as sociodemographic characteristics and risk factors were considered, only women with complex activity limitations remained significantly more likely than nondisabled women to be diagnosed with cancer [18]. It could be argued that those reporting difficulties with the different studied physical activities, which are basic daily movements, have a more advanced disability state. Interestingly, a systematic review and meta-analysis showed that physical function outcomes (handgrip strength, gait speed…) were significantly associated with mortality in cancer patients [19]. These results could suggest that physical function is a relevant factor in the cancer experience and should be one of the main targets in the management of patients.

In this study, we observed that participants with difficulties lifting or carrying weights had the highest significant odds for cancer, closely followed by reporting difficulties pulling or pushing large objects. In addition, we observed an inverse association between handgrip strength and cancer diagnoses. Muscle strength might be a crucial component of health-related fitness in cancer patients as it plays an independent role in its prevention and treatment [20], being associated with better results after major abdominal surgery [21, 22]. In fact, upper and lower limb muscle strength is inversely and independently associated with death from all causes and cancer in men [23]. Moreover, Versteeg et al. [24] found that patients with higher muscle strength treated with chemotherapy had longer overall survival than patients with lower muscle strength in a sample of 103 patients with advanced cancer. A systematic review with a large sample of patients with various cancer types, stages, and treatment modalities estimated that the overall prevalence of sarcopenia in cancer patients before starting the treatment was 38.6% [25]. Furthermore, sarcopenia was associated with poor outcomes during cancer treatment, like postoperative complications, chemo-therapy-induced and dose-limiting toxicity and overall, relapse-free, and progression-free survival [25]. These findings highlight the importance of muscle strength and muscle function in this population.

In this study, reduced lower limb physical function such as difficulties stooping, kneeling, or crouching, getting up from a chair and climbing one or several flights of stairs was associated with higher odds of cancer diagnoses. Since these activities also are partially dependent on muscle strength, our results seem logical and could be partially explained at the above paragraph. Similar to our results, a previous study [26] in which cancer patients were assessed using standardized questionnaires related to their performance in different activities of daily living, reported that some of the activities in which patients reported having difficulties (either due to the need to extra time, greater effort, or risk) were going outside, moving between rooms using stairs, and sitting/getting up from a chair. Furthermore, they found no significant gender difference in self-reported quality of activities of daily living performance [26]. Moreover, some of these activities, such as stair climbing, have been used as an economical and widely applicable tests [27], able to explain physical function and recovery in cancer patients [28].

Importantly, we also observed an association between difficulties walking 100 m and cancer diagnoses in the present study. This is critical for patients who have difficulties getting up from a chair or climbing a flight of stairs since the movement is based mainly on the lower limb physical function. Walking is an action that requires postural alignment, muscular strength, perceived limit of stability, sensory integration, anticipatory and reactive postural reactions, and cognition [29]. Walking difficulties are rarely recorded in outpatient oncology settings [30]. However, in a cross-sectional study, authors found that among survivors of four major cancers (breast, colorectal, lung, and prostate cancer) who were 1–5 years after diagnosis, 28–45% reported having difficulty walking or maintaining balance [31]. Impairment in balance and walking is a major risk factor of fall [32] and having cancer increases the risk of in-hospital mortality after an injury from a trauma [33]. Therefore, these patients should receive adequate treatment and especially follow-up.

Strengths of the present study comprise the use of data from a large, reliable, and representative dataset, from a publicly available institutional repository. Furthermore, an important exposure variable such as handgrip strength was objectively measured.

The data from this study suggest that there is an association between difficulties in daily physical activities and handgrip strength with the diagnosis of cancer. Although the direction of the association is not clear, on one hand, we must address the limitations in daily physical activity in patients who suffer or have suffered from cancer, so strength training could be an essential tool capable of increasing strength and muscle function. On the other hand, health authorities should implement detection (ideally at primary care) and monitoring policies to control people who have physical difficulties and periodically facilitate evaluations.

We also acknowledge several limitations for the present study. Importantly, statistical associations between exposure and outcome variables were observed; however, we cannot make causal inferences, mainly due to the temporal ambiguity that arises when simultaneously measuring exposure and disease. Thus, the direction of the association cannot be established. Further investigation may elucidate the direction of the observed association. Also, several estimations with low significant ORs may not be clinically meaningful. Moreover, the absence of the date and detailed information on cancer diagnosis such as type of cancer or cancer stage is a major limitation, which may entail certain degree of attenuation of the observed associations (i.e., those experiencing one specific type of cancer or cancer stage may be more affected than others with different specific cancer type and/or cancer stage). Furthermore, there is a risk of both recall and misclassification bias due to self-reporting cancer diagnosis, which might be exacerbated with the age. The low number of participants self-reporting cancer diagnosis is low (4.8%), which may influence estimates in specific categories with fewer cancer cases. Finally, the possibility of a residual confounding bias concerning variables not included in the model is still plausible, although the observed associations remained robust even when submitted to sensitivity analyses.

## Conclusions

Having only one or more difficulties in daily physical activities as well as lower levels of handgrip strength is positively associated with cancer diagnoses. Those with difficulties lifting or carrying weights over 5 kilos or having difficulties in two or more daily physical activities showed especially remarkable associations. The present data can be used to implement interventional strategies in those with the underscored difficulties. Our results warrant further investigation to determine the causality of the observed associations.

### Supplementary Information

Below is the link to the electronic supplementary material.

Supplementary file1 (DOCX 22 KB)

## Data Availability

The data are publicly available in http://www.share-project.org/home0.html upon request.
