# Supplementary material for: Association of difficulties in daily physical activities and handgrip strength with cancer diagnoses in 65,980 European older adults
Source: Aging Clin Exp Res. 2023 Oct 27;35(12):2971–8. doi: 10.1007/s40520-023-02577-7 (PMC10721652; doi:10.1007/s40520-023-02577-7)
Supplement: Supplementary file 1 — Supplementary file1 (DOCX 22 KB) [file 40520_2023_2577_MOESM1_ESM.docx]

| **Table S1. Associations between different physical movements and current or prior cancer (N=42,761).** | | | | | | | |
| --- | --- | --- | --- | --- | --- | --- | --- |
|  |  | Model 1 | | Model 2 | | Model 3 | |
|  |  | OR | 95%CI | OR | 95%CI | OR | 95%CI |
| Difficulties  walking 100 m |  |  |  |  |  |  |  |
|  | No | ref | ref | ref | ref | ref | ref |
|  | Yes | 1.10 | 0.84-1.45 | 0.87 | 0.66-1.13 | 1.65 | 1.49-1.83 |
| Difficulties sitting two hours |  |  |  |  |  |  |  |
|  | No | ref | ref | ref | ref | ref | ref |
|  | Yes | 1.38 | 1.02-1.87 | 0.99 | 0.73-1.35 | 1.68 | 1.51-1.86 |
| Difficulties getting up from a chair |  |  |  |  |  |  |  |
|  | No | ref | ref | ref | ref | ref | ref |
|  | Yes | 1.64 | 1.27-2.11 | 1.16 | 0.92-1.48 | 1.60 | 1.46-1.74 |
| Difficulties climbing one flight of stairs |  |  |  |  |  |  |  |
|  | No | ref | ref | ref | ref | ref | ref |
|  | Yes | 1.53 | 1.06-2.21 | 1.17 | 0.80-1.72 | 1.56 | 1.41-1.72 |
| Difficulties climbing several flights of stairs |  |  |  |  |  |  |  |
|  | No | ref | ref | ref | ref | ref | ref |
|  | Yes | 1.91 | 1.45-2.52 | 1.34 | 1.00-1.80 | 1.78 | 1.63-1.91 |
| Difficulties stooping, kneeling, or crouching |  |  |  |  |  |  |  |
|  | No | ref | ref | ref | ref | ref | ref |
|  | Yes | 1.53 | 1.22-1-93 | 1.04 | 0.83-1.30 | 1.52 | 1.41-1.65 |
| Difficulties reaching or extending arms above shoulder |  |  |  |  |  |  |  |
|  | No | ref | ref | ref | ref | ref | ref |
|  | Yes | 1.90 | 1.38-2.62 | 1.38 | 1.01-1.87 | 1.82 | 1.63-2.03 |
| Difficulties pulling or pushing large objects |  |  |  |  |  |  |  |
|  | No | ref | ref | ref | ref | ref | ref |
|  | Yes | 1.94 | 1.54-2.45 | 1.38 | 1.11-1.73 | 1.96 | 1.80-2.14 |
| Difficulties lifting or carrying weights over 5 kilos |  |  |  |  |  |  |  |
|  | No | ref | ref | ref | ref | ref | ref |
|  | Yes | 1.91 | 1.48-2.46 | 1.33 | 1.04-1.70 | 2.07 | 1.91-2.25 |
| Difficulties picking up a small coin from a table |  |  |  |  |  |  |  |
|  | No | ref | ref | ref | ref | ref | ref |
|  | Yes | 1.03 | 0.71-1.48 | 0.78 | 0.53-1.14 | 1.48 | 1.26-1.75 |
| Handgrip (kg) tertiles |  |  |  |  |  |  |  |
|  | Third 3 | ref | ref | ref | ref | ref | ref |
|  | Third 2 | 0.94 | 0.71-1.26 | 0.89 | 0.66-1.18 | 1.16 | 1.05-1.30 |
|  | Third 1 | 1.08 | 0.78-1.48 | 0.89 | 0.65-1.22 | 1.32 | 1.16-1.51 |
| Several movement difficulties (2 or more) |  |  |  |  |  |  |  |
|  | No | ref | ref | ref | ref | ref | ref |
|  | Yes | 2.27 | 1.67-3.07 | 1.45 | 1.04-2.02 | 2.01 | 1.85-2.17 |
| Model 1. Survey and weighted adjusted for age, sex, body mass index, and country.  Model 2. Survey and weighted adjusted for Model 1 + multimorbidity (current or previously experiencing 2 or more of the following conditions: heart attack, hypertension, cholesterol, stroke, diabetes, chronic lung disease, cancer, stomach or duodenal ulcer, Parkinson, cataracts, hip fracture, other fractures, Alzheimer, emotional disorder, rheumatoid arthritis, osteoarthritis, or kidney disease)  Model 3. Imputed missing values (multiple imputation) and adjusted for Model 1 + education (ISCED 1997 classification) + physical inactivity + alcohol consumption + fruits and vegetables consumption + current smoking habit.  OR Odds Ratio  CI Confidence Interval  ref Reference  Note: Missing values imputed for Model 3 corresponds to 30% of the sample. | | | | | | | |

**Supplementary informational for additional variables.**

Education was self-reported by participants and then coded using the 1997 version of the International Standard Classification of Education (ISCED). Physical inactivity was assessed through two questions: “How often do you engage in vigorous physical activity such as sports, heavy housework, or a job that involves physical labour”, and “How often do you engage in activities that require a moderate level of energy such as gardening, cleaning the car, or doing a walk?”. Participants selecting the option of “Hardly ever, or never” in the two questions were considered physically inactive. Alcohol consumption was determined through the question: “Did you consume at least one alcoholic beverage the last 7 days?”, and potential answers comprised “Yes”, “No”, “Refusal” or “Don’t´ Know”. Current Fruits and vegetables consumption was examined with the following question: “In a regular week, how often do you consume a serving of fruits or vegetables?”, and potential answers comprised the following options: “Refusal”, “Don’t´ Know”, “Everyday”, “3-6 times a week”, “Twice a week”, “Once a week”, and “Less than once a week”. Current smoking habit was assessed using the following question: “Do you smoke at the present time?” and the potential answers were “Yes”, “No”, “Refusal” or “Don’t´ Know”.
